# High-Precision In Situ ^87^Sr/^86^Sr Analyses through Microsampling on Solid Samples: Applications to Earth and Life Sciences

**DOI:** 10.1155/2018/1292954

**Published:** 2018-04-22

**Authors:** Sara Di Salvo, Eleonora Braschi, Martina Casalini, Sara Marchionni, Teresa Adani, Maurizio Ulivi, Andrea Orlando, Simone Tommasini, Riccardo Avanzinelli, Paul P. A. Mazza, Sandro Conticelli, Lorella Francalanci

**Affiliations:** ^1^Dipartimento di Scienze della Terra, Università degli Studi di Firenze, via Giorgio La Pira 4, 50121 Firenze, Italy; ^2^C.N.R., Istituto Geoscienze e Georisorse, U.O. di Firenze, via Giorgio La Pira 4, 50121 Firenze, Italy

## Abstract

An analytical protocol for high-precision, in situ microscale isotopic investigations is presented here, which combines the use of a high-performing mechanical microsampling device and high-precision TIMS measurements on micro-Sr samples, allowing for excellent results both in accuracy and precision. The present paper is a detailed methodological description of the whole analytical procedure from sampling to elemental purification and Sr-isotope measurements. The method offers the potential to attain isotope data at the microscale on a wide range of solid materials with the use of minimally invasive sampling. In addition, we present three significant case studies for geological and life sciences, as examples of the various applications of microscale ^87^Sr/^86^Sr isotope ratios, concerning (i) the pre-eruptive mechanisms triggering recent eruptions at Nisyros volcano (Greece), (ii) the dynamics involved with the initial magma ascent during Eyjafjallajökull volcano's (Iceland) 2010 eruption, which are usually related to the precursory signals of the eruption, and (iii) the environmental context of a MIS 3 cave bear, *Ursus spelaeus*. The studied cases show the robustness of the methods, which can be also be applied in other areas, such as cultural heritage, archaeology, petrology, and forensic sciences.

## 1. Introduction

In situ radiogenic isotope determinations with microscale resolution, especially of Sr, can represent a powerful tool in different fields of geological and life sciences. In particular, this technique is nowadays one of the most important methods for the investigation and interpretation of magmatic processes, as well as of environmentally-induced responses of terrestrial mammals; it has the potential to greatly enhance our understanding of not only volcanic systems and the related magma genesis and evolution, but also of the physiological mechanisms behind specific organic adaptations.

In situ ^87^Sr/^86^Sr provides significant data on the (i) source heterogeneities of magmas, (ii) crystallization histories within shallow level magmatic reservoirs, and (iii) magma residence times prior to eruptions (e.g., [1–10]). Since crystals record changes occurring in the environment in which they grow (e.g., [7, 11–15]), isotopic investigations at grain and subgrain scales on rock-forming minerals provide information on mineral-whole rock equilibria that constrain the magmatic processes occurring during magma evolution (e.g., mixing, mingling, crystals recycling, crustal contamination, or metasomatism). In addition, variation of Sr-isotope composition from core to rim within the same crystal can shed light on the complex pre-eruptive history of active volcanoes. Therefore, combining micro-(small-scale) isotope data with textural and petrographic data provides significant information on crystals residence time, magma production rates, and recharge dynamics (e.g., [5–8, 16–18]).

Sr-isotopic investigation has recently gained popularity in other fields, such as archaeology, anthropology, biology, cultural heritage, environmental and food studies, forensics, life and medical sciences, and palaeontology. (e.g., [19–38]). ^87^Sr/^86^Sr on tooth enamel and bone tissues gained particular attention over the last years in life studies, archaeology, palaeontology, and forensic sciences (e.g., [39–48]). In situ analyses of ^87^Sr/^86^Sr using laser ablation and multicollector-inductively coupled plasma mass spectrometry (LA-MC-ICPMS) for tooth enamel, bones, and rocks were developed in the early new millennium but have met variable success (e.g., [49–53]). At the same time, microscale sampling through drilling and micro-Sr isotope analyses by thermal ionisation mass spectrometry (TIMS) was also developed on geological materials (e.g., [6, 18, 54]). As compared with mechanical microdrilling plus TIMS procedures, LA-MC-ICPMS has the advantage of significantly reducing the time of analyses, but at the expense of precision of the measured isotope ratio; this is generally due to smaller Sr signals and the need to correct for isobaric interferences (e.g., [16, 53]).

We present a detailed protocol for in situ sampling through microdrilling, Sr purification, and thermal ionisation mass spectrometer (TIMS) high-precision determinations of small amounts of Sr (<10 ng) in both biological and geological materials at the same error levels. In situ ^87^Sr/^86^Sr analysis is presented in three case studies which deal with the following: (i) plagioclase crystals from Nisyros volcano (Greece), (ii) glassy matrix of single glassy clasts from the 2010 eruption of the Eyjafjallajökull volcano (Iceland), and (iii) bones and teeth from fossil remains of the *Ursus spelaeus*. In these three examples, the in situ Sr-isotope approach permits to constrain petrological and volcanological processes and to effectively outline the life habits and habitat exploitation of extinct living species. The three presented cases aim to show the high potential of the methodology, validating its wide-scale applicability in many other areas, such as cultural heritage, archaeology, and forensic sciences.

## 2. Materials and Methods

Technological improvements on mass spectrometer and microsampling devices allow researchers to collect and analyse small amounts of sample (few micrograms of sample, containing nanograms of Sr) with no loss of precision in the isotopic determination. In situ analyses have many important advantages over more typical ones on bulk samples. In geological applications, it allows to preserve the textural information and thus to combine it with the isotopic and geochemical composition of specific portions of the samples. In archaeology and palaeontology, this method has the advantage of minimising the damage and/or destruction of samples, thereby leaving significant amounts available for further applications.

The procedure consists in three main stages: (i) in situ sampling through microdrilling, (ii) sample digestion and purification of the element of interest, in our case Sr, and (iii) measurement of the isotope ratios (i.e., ^87^Sr/^86^Sr) through thermal ionisation mass spectrometry (TIMS). Our Radiogenic Isotope Laboratory at the Department of Earth Sciences of the Florence University is equipped with a modern MicroMill™ grinder device, an ultraclean laboratory (“Class 1000”) for microsamples digestion and elemental separation and a thermal ionisation mass spectrometer (ThermoFinningan™ Triton-Ti®) for isotopic measurements.

Compared to classic, bulk sample analyses (generally measured on 100–150 ng of Sr [55]), small-sample analysis (typically 5 to 10 ng of Sr) has the drawback of being more exposed to contamination from Sr alien to the sample. In situ micro-Sr measurements therefore require continued testing of laboratory blanks during the whole analytical procedure.

### 2.1. In Situ Sampling

#### 2.1.1. The Microdrilling Device

**Figure 1:**
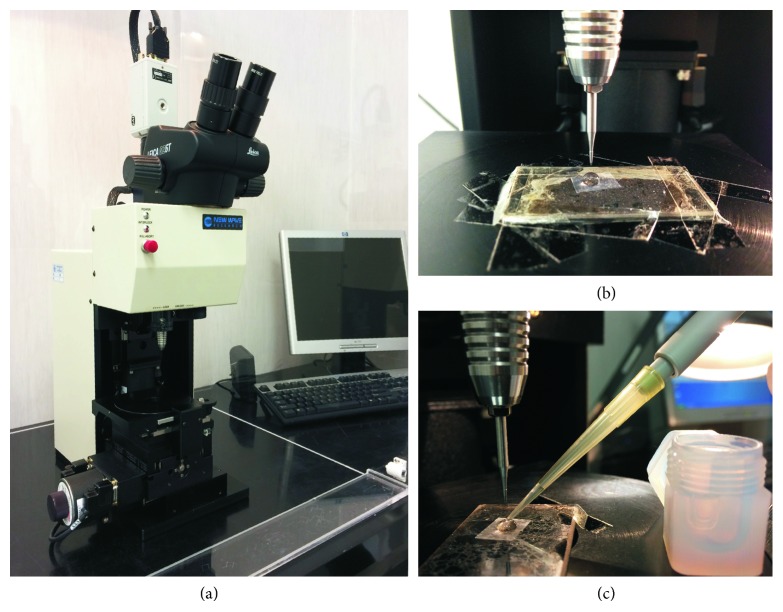
(a) The New Wave-Merchantek Micromill device operating at the Department of Earth Sciences–University of Firenze; (b) image of a petrographic polish thick section fixed on the sample stage under the tungsten carbide drilling bit that is locked into the milling chuck. A Milli-Q droplet constrained by the Parafilm is placed on the section in order to collect the powder during the drill; and (c) sample slurry recovery from the drilled surface into the digestion beaker.

**Figure 2:**
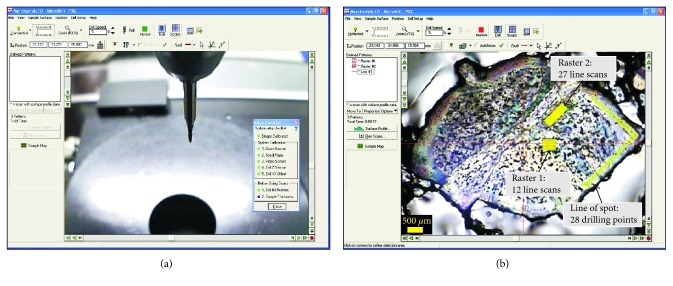
(a) Live image of the sample stage and drill bit shown by the digital camera placed next to the milling chuck; (b) microscope view image showing a zoned plagioclase crystal with drilling pattern. Two rasters (in yellow) are set up to drill the plagioclase core, whereas a line of spot is set up for the rim microdrilling.

Microsampling on minerals, glasses, and tooth enamel reported in this paper was performed using a microdrilling device capable of high resolution milling (New Wave-Merchantek MicroMill™, https://www.esi.com/products/laser-processing/milling/micromill/). The MicroMill (Figure 1) combines a binocular microscope (with 6.7x–40x magnifying power) with remotely controlled submicron stage resolution and positional accuracy and a real-time video observation (at 3x digital magnification). It includes a low-eccentricity high-torque milling chuck, with variable speed (1,200–35,000 rpm), wherein a tungsten carbide or diamond-tipped bit is fixed, and an automated high-precision sliding stage on which the sample is loaded (Figure 1). The open stage architecture can accommodate thin sections or mounts and also larger solid samples such as bones, shells, ceramics, and plastics. The plug where the bit is hosted moves with adjustable speed for both spin and vertical movement, along the Z direction. The stage moves along the X-Y direction with a precision of 1 *μ*m and maximum shift of 5 cm, allowing a high spatial resolution to the micron scale. This yields high spatial resolution, to the micron scale, and allows small-size sampling (i.e., a few *μ*g of powder). The digital camera, placed next to the milling chunk (Figure 1), shows a live image of the stage and of the bit position (Figure 2(a)). Using the microscope position mode (scope position), the optical image of the sample can be monitored on the PC screen prior to and after the sampling (Figure 2(b)).

The device allows in situ microsampling on several types of solid materials such as rocks, minerals, glasses, plastics, bone tissues, ceramics, metals, and alloys. The designed software package also allows performing different milling patterns such as holes arranged randomly, lines, or rasters (Figure 2(b)). Fine adjustment of milling velocity helps the microsampling of solid materials with different hardness.

Microsampling on geological (e.g., rock, minerals, and glasses) and archaeological materials (glasses, ceramics, teeth, and bones) is performed using either thick polished sections (some 100 *µ*m thick) or sample mounts (e.g., small chunks, chips, fragments of bones, and teeth). In our case studies, we used thick, polished sections for mineral and volcanic glasses and mounts for the teeth; both were fixed on the stage using either a double-sided adhesive tape or hot glue, to avoid sample displacement during milling. Thick, polished sections are preferable for geological samples because they permit to characterise the petrographic features of the samples and thus to perform the microsampling according to their textural properties.

#### 2.1.2. Milling Procedure and Sample Collection

A droplet of Milli-Q® water is placed with a micrometric pipette on the selected area prior to milling; this is performed by sticking a small punched square of warmed-up Parafilm™ on the sample surface (Figure 1). The water droplet retains the powder produced by the milling, which can then be easily collected by pipetting; it has also the effect of cooling the microdrill bit while milling. Before each drilling session, the drill bit is ultrasonically cleaned with pure ethanol and then rinsed with Milli-Q water.

**Figure 3:**
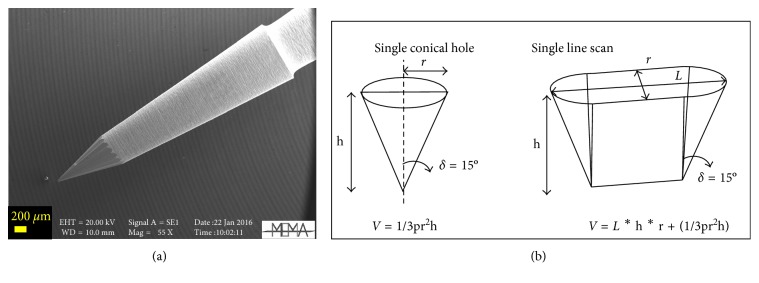
(a) SEM imaging of a tungsten carbide drill bit used for milling: the tip angle is 30° and geometrical shape of (b) a single conical hole and single line scan.

The instrument software provides different drilling patterns and depths: single or multiple independent holes, spot lines, grids, line scans, or rasters (Figure 2(b)). Milling spot lines (or grids) are more accurate, but more time-consuming; they were used for the geological material (crystals and volcanic ashes) which requires more precise spatial resolution between the different zones of the same crystal or between the thin films of glass. Milling failure, such as crystal breaking, was prevented by setting a slow scan speed and splitting the milling into two or more steps. Line scans, which are performed faster but less accurately, were used for drilling the teeth. The number of points, lines, or rasters to be milled (which accounts for the amount of Sr to be collected) need to obtain a sufficient quantity of Sr for the TIMS measurements and can be calculated based on (i) the Sr content of the sample (independently determined by LA-ICPMS), (ii) the geometry of the drill bit, and (iii) the drilling pattern and the depth. Tips of different size and shape can be used for the drilling; therefore, the volume of material actually removed from different depths of a single hole or at different depths and lengths of a single line needs to be carefully calculated. The tungsten carbide mill bits supplied with the microdrill device (Komet–Brassler), have conical shape with an angle of 30° (Figure 3). The volume removed during each drilling is equivalent to that of the conical tip and dependent on the geometry of the drilling pattern, as well as on the specific depth (Figure 3). The minimum amount of sample that needs to be drilled depends also on the total procedural blank, which should be at least two orders of magnitude lower than the total amount of Sr collected from the sample.

After milling was completed, the sample slurry was collected with a micropipette in a PFA beaker and then transferred in the clean lab for sample digestion and elemental purification. The blanks of the milling procedure were determined by keeping the drill bit tip into a Milli-Q water droplet on the sample surface (accurately cleaned before use) for as long as the average sampling time; the droplet was then processed as an ordinary sample. The amount of Sr in the blank was then determined through isotope dilution, by adding a single-spike solution (enriched in ^84^Sr).

### 2.2. Sample Dissolution and Sr Purification

The purification of the element of interest, in our case Sr, is crucial to obtain high-precision isotopic measurements for at least two reasons. First, it avoids isobaric interferences on the masses that will be analysed by mass spectrometry; in the specific case of Sr isotope measurements, even a small amount of ^87^Rb will add to ^87^Sr, yielding an overestimate of the ^87^Sr/^86^Sr ratio. Secondly, the presence of other elements of the matrix will compete with Sr during the thermal ionisation process, reducing the Sr signal and thus yielding less accurate measurements. The possibility of collecting and processing the sample for the purification of the element of interest is one of the major advantages of the method presented here over other methodologies, which do not achieve the same degree of accuracy and precision. The LA-MC-ICPMS methods allow faster data acquisition and higher sample throughput than mechanical microdrilling plus TIMS procedures, thanks to the possibility of introducing the samples directly into the mass spectrometer without chemical separation. On the other hand, LA-MC-ICPMS measurements require careful monitoring and corrections to minimize isobaric interferences in order to achieve suitable analytical accuracy and precision (e.g., [49, 53, 54, 56]).

**Figure 4:**
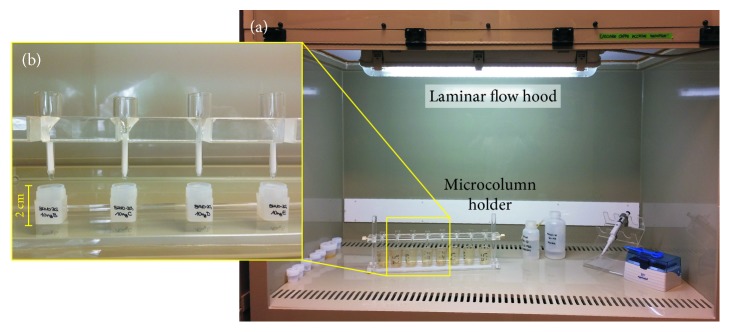
(a) Image of the laminar flow hood used for chemical processing and Sr separation of microdrilled samples; (b) image of quartz microcloumns filled with approximately 140 *µ*l of specific chromatographic resin (Eichrom Sr-Spec, 100–150 *µ*m) with high Sr-recovery efficiency, for Sr element extraction.

Powder digestion and Sr purification were carried out in our ultraclean laboratory (“Class 1000”) aiming at the following: (i) optimising the separation of Sr and Rb to avoid interference of ^87^Rb with ^87^Sr, (ii) purifying the Sr collection form all the matrix analytes, (iii) maximising the yield of the columns during the chromatographic purification, and (iv) preserving low procedural blanks. Sample digestion was performed by sequential HF-HNO_3_-HCl as described in [55]. Chromatographic Sr purification was performed using Eichrom® Sr-Spec™ resins (100–150 *µ*m) in quartz microcolumns (0.14 ml volume; Figure 4). Matrix elements were flushed out through elution with 14 column volumes of 3 N HNO_3_. Sr was then collected in Milli-Q (13 column volumes). The collected Sr fractions were further treated with concentrated HNO_3_ and H_2_O_2_ (fluxing at 150°C on a hotplate) to remove any organic residue. After this final step, samples were diluted in HNO_3_ (10 vol.%) and were finally ready for loading on filaments for mass measurements. The whole analytical procedure was performed with acids of ultra-pure quality.

In order to thoroughly assess the contamination levels, we measured two types of blanks, one considering only the amount of Sr deriving from the chemical digestion and Sr separation, the other accounting for the whole procedure, including the drilling process, as described in the previous section. The results were 17 ± 6 (1 SD, *n* = 12) and 38 ± 19 pg (1 SD, *n* = 16), respectively, over a 14-month period (Table 1), thus allowing sampling as low as 4 ng of Sr for isotope analysis.

### 2.3. ^87^Sr/^86^Sr Measurements on TIMS

**Figure 5:**
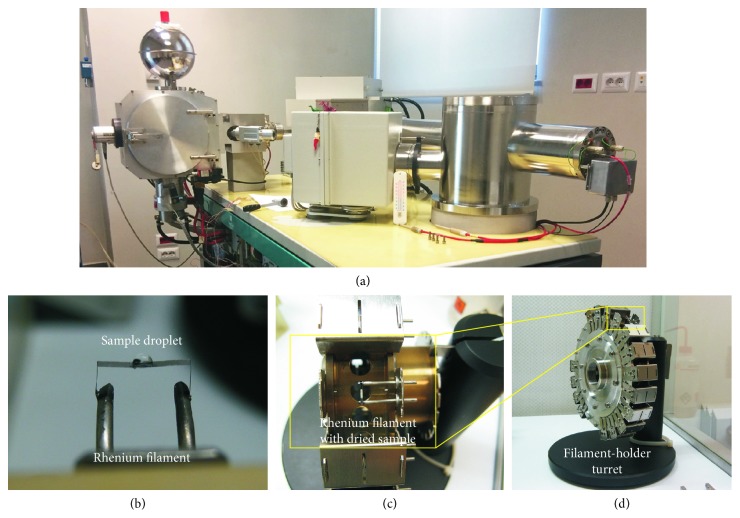
(a) The ThermoFinningan Triton-Ti multicollector, thermal ionization mass spectrometer (TIMS) at the University of Firenze; (b) single-rhenium filament with a sample droplet on top; (c) detail of a single filament, holding a dried sample loaded on a sample holder turret that can host 21 filament positions; (d) the turret is ready for the installation into the thermal ionization mass spectrometer.

Sr isotope ratios were determined using a multicollector, thermal ionization mass spectrometer (TIMS: ThermoFinningan Triton-Ti™) (Figure 5), equipped with nine moveable collectors, which allow to simultaneously detect all the natural masses of Sr (^84^Sr, ^86^Sr, ^87^Sr, and ^88^Sr). The mass of ^85^Rb was also measured to monitor possible ^87^Rb interference, but it was always lower than the detection limit of the instrument, confirming the quality of the separation procedure described above. A detailed description of instrumental characteristics and performances are given in [55], along with standardised routine, measuring conditions, and setting for normal-sized samples (100–150 ng of Sr). Instrumental mass bias (e.g., [57–59]) was corrected to the natural value of ^86^Sr/^88^Sr = 0.1194 using an exponential law (e.g., [55, 59]).

The most critical aspects of measuring small-size samples are related to (i) the procedure of sample loading onto the filaments and (ii) the measurement mode (i.e., static versus multidynamic, e.g., [55, 59]). Both are very important to maximise the Sr signal during the measurements, to balance the analysis time and the analytical errors that are a function of sample size.

The measurement protocol was tested by replicate analyses of an international certified standard (NIST-SRM987), properly diluted to attain sample sizes (5 to 10 ng Sr) comparable to those of the microsamples. Then, we tested the whole procedure, from in situ sampling (<10 ng of Sr) to isotope measurement, on the international glass reference sample BHVO-2G. BHVO-2G reference sample is a synthetic basaltic glass (provided by USGS) obtained by melting the BHVO-2 powder collected from a Hawaiian lava flow. The glassy slices are supplied in epoxy resin mounts (https://crustal.usgs.gov/geochemical_reference_standards/microanalytical_RM.html).

#### 2.3.1. Sample Loading onto Filament

The Sr fraction collected from the columns was dissolved into 1 *µ*l HNO_3_ 10 vol.% and loaded on single Re filaments under a horizontal laminar flow hood. Due to the small amount of Sr available, it is important to confine the sample on the smallest possible area on the filament, so that the whole loaded sample can be ionised at the same time from a single spot. To attain this, a thin layer of Parafilm™ was melted at both sides of the filament surface, to prevent any spreading of the solution, leaving a small gap (1 mm) for the droplet at the center on the filament (Figure 5). The loading was performed by sandwiching the sample between two 0.5 *µ*l drops of TaCl_5_ activator solution [55] and 0.5 *µ*l of H_3_PO_4_ solution (6 vol.%), respectively. TaCl_5_ activator was added for enhancing Sr ionisation efficiency and H_3_PO_4_ for stabilising Sr isotope fractionation during measurement. All the solutions (i.e., activator, sample, and H_3_PO_3_) were slowly dried by passing a current on the filament, which was increased at the end of the procedure until the filament starts glowing. The loaded filaments were then placed on the filament-holder turret and then inserted into the mass spectrometer (Figures 5 and 5).

#### 2.3.2. Measurement Procedure Reproducibility and Accuracy

The measurement routine was established to obtain the best internal and external precisions, and the accuracy, on ^87^Sr/^86^Sr, was achieved by experimentally comparing runs performed in *static* versus *dynamic* conditions at a variable number of cycles and integration times. A detailed description of the *static* and *dynamic* methods is provided in [55, 59]. In brief, the static mode consists of simultaneous measurements of all isotopes in a single “jump,” so that the magnetic field remains static and the masses always hit the same detectors (Table 2). *Static* measurements have the advantage of considerably reducing the acquisition time in comparison with the *dynamic* mode, which becomes important when little amount of Sr is available, as dealing with small samples. The main limitation of this method is related to the uncertainty on the Faraday cup efficiency and on the drift of the electronics (i.e., the amplifiers). The Triton-Ti is equipped with a virtual amplifier, which enables a variable connection between amplifiers and Faraday cups and allows a complete switching between amplifiers and cups during a single measurement. However, the virtual amplifier is not able to correct for the different Faraday cup efficiency and its variation with time.

In contrast, the *dynamic* (or *multidynamic*) mode is a peak-jumping procedure where a number of different cup configurations are employed for determining a single isotopic ratio (Table 2). This means that each isotope beam is measured sequentially in different Faraday cups, so that two ^87^Sr/^86^Sr_double_ values (Table 2) can be calculated without cup efficiency biases and drifts of the electronics (Table 2). The two ^87^Sr/^86^Sr_double_ values are then geometrically averaged to obtain a single ^87^Sr/^86^Sr_triple_ value.

The best configuration for *static* mode measurements was found by measuring 300 cycles with an integration time of 8 s, which corresponds to a total measuring time of about 35 minutes for each sample. For *dynamic* mode measurements, we performed 120 cycles (each including 3 magnetic jumps), with 8 s of integration time and an idle time of 3 s between the different jumps, for a total of 70 minutes for each sample.

In both *static* and *dynamic* methods, the filament was slowly warmed up, for a total of about 45 minutes, to stabilise the ion emission until the suitable intensity is achieved. During the heating, the beam was accurately optimised by peak-centering and focusing. The optimal beam intensity for the measurement varied from *static* to *dynamic* mode, with higher intensity allowed by the shorter *static* (3–3.5 V on ^88^Sr) mode with respect to *dynamic* (1.5–2 V on ^88^Sr) mode, which instead requires maintaining a stable beam, owing to the longer duration of the measurement.

**Figure 6:**
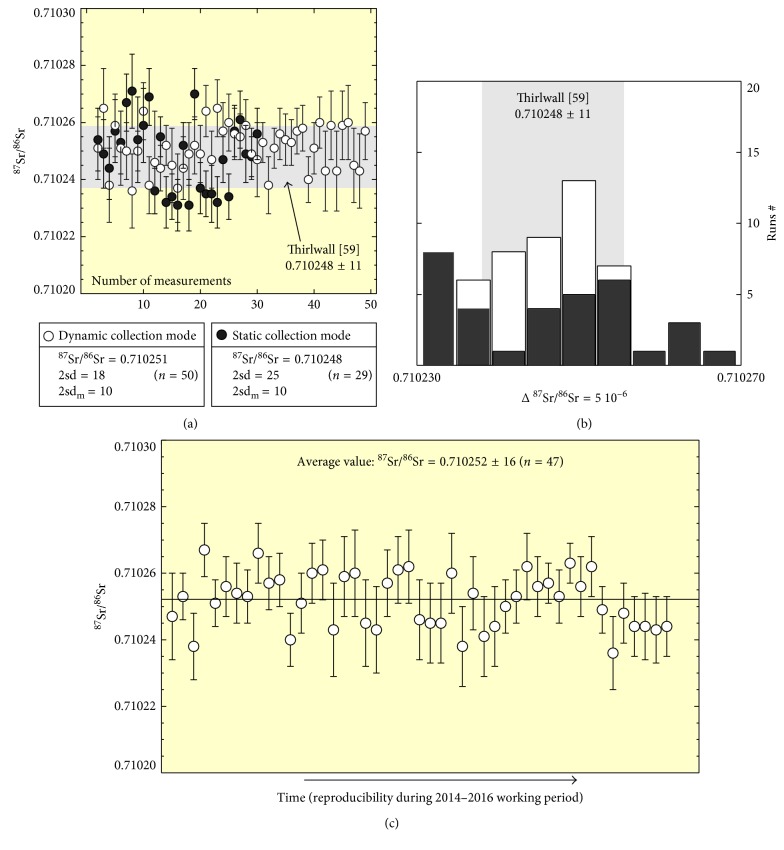
(a) Reproducibility and accuracy for repeated measurements of ^87^Sr/^86^Sr on 12 ng load size of NSIT-SRM987 standard material for static versus dynamic collection mode over a period of 10 months. Each single measurement is plotted with the relative error bars. The grey-shaded field shows the [59] recommended value range. (b) Distribution of the ^87^Sr/^86^Sr values measured all over the period of analysis with the static and dynamic collection mode: the static collection mode (black filled columns) shows a worst external reproducibility than that obtained in the dynamic mode (open columns); indeed the dynamic mode gives measurements that reasonably fit a Gaussian distribution pattern with the more representative ^87^Sr/^86^Sr values centered within the [59] recommended interval (grey shade fields); (c) reproducibility and accuracy trend of ^87^Sr/^86^Sr 12 ng load size of NSIT-SRM987 measured in the dynamic collection mode throughout the setup period (from 2014 to 2016).

The results are shown in Figure 6 and Table 3. *Static* and *dynamic* mode measurements on NIST-SRM987 reference samples (10 ng of Sr measured) yielded ^87^Sr/^86^Sr average values of 0.710247 ± 0.000026 (2 SD, *n* = 30) and 0.710251 ± 0.000018 (2 SD, *n* = 51), respectively, with internal precisions of 13 ppm (2 SE) and 16 ppm (2 SE), respectively. Both values are within the recommended reference value for NIST-SRM987 (^87^Sr/^86^Sr = 0.710248 ± 0.000011; Figures 6(a) and 6(b) and Table 3 [59]). *Static* measurement reduced the experimental time but showed a worse external reproducibility than that obtained in *dynamic* mode (Figures 6(a) and 6(b)), yet maintaining similar internal precision. Therefore, the *dynamic* mode was chosen for the experimental work both on the international glass standard BHVO-2G and on the unknown samples. Further measurements of SRM987 in the *dynamic* mode were performed after the initial testing, along with the studied samples, yielding consistent results (^87^Sr/^86^Sr = 0.710252 ± 0.000018, 2 SD, *n* = 47; Figure 6(c)).

Results on the BHVO-2G are reported in Table 3. The results were also compared to standard measurements (150 ng of Sr) on the BHVO-2 powder reference sample. BHVO-2G versus BHVO-2 results found for micro- and normal-size samples, respectively, are well within the internal analytical error (Table 3) and in agreement with the reference values reported in [60, 61] for bulk powder (i.e., standard BHVO-2). The significantly larger standard deviation of the microdrilled BHVO-2G measurements, with respect to both micro-Sr SRM987 and BHVO-2 powder data, is likely partly related to small isotopic heterogeneities of the glass standard. The few available micro-Sr data on the same sample provide similar averages and reproducibility of our data [54, 62] (Table 3).

Comparing our results with LA-MC-ICPMS data is more difficult; in fact, the latter vary largely depending on the material used for the analyses. External reproducibility obtained with LA-MC-ICPMS on material with high Sr contents and low Rb/Sr (e.g., apatite [49, 53], marine shells, and synthetic plagioclase [16]) is comparable or slightly worse than that attained with our method; yet, small but significant differences in accuracy have been reported [53]. On the other hand, the internal and external reproducibility worsen significantly (e.g., by a factor 5 to 10 in [16]) in materials with low Sr and high Rb/Sr.

In summary, the method presented here generally provides more accurate and precise results than LA-MC-ICPMS, independently on the nature of the analysed material, despite being more time-consuming. It is therefore suitable for a wider range of applications.

## 3. Applications

In this section, we report three case studies, two of which were previously published [8, 10], as examples of possible applications of the presented methodology in different fields of science. Indeed, in the last decade, the in situ isotope microsampling approach has been used and applied in many pilot studies in a wide range of research fields, including, among others, palaeoenvironmental and palaeoecologic reconstructions (i.e., [53, 63]) and climate changes (i.e., [64]).

In the three presented case studies, the samples were thoroughly characterised both texturally (optical microscope and SEM) and chemically (electron micro probe analyses) before drilling. The strontium element concentrations (in ppm) in all the samples was then determined through LA-ICPMS.

### 3.1. Micro-Sr Isotope in Minerals

Rock-forming minerals in igneous rocks display variable chemical composition depending on several processes and parameters such as (i) the physicochemical conditions of the magmas, (ii) open system processes (e.g., magma mixing and mingling), and (iii) recycling of cumulated crystals triggered by new arrivals of magma within crustal reservoirs. Radiogenic isotope ratios in minerals, or portions of them, can be used as a petrogenetic “DNA” to record the history of the magma reservoir (*crystal isotope stratigraphy*, e.g., [7]) and their evolution within the crust (e.g., [1, 5, 7, 8, 65–67]). Combining in situ Sr-isotope fingerprints with other approaches, such as textural evidences and crystal size distribution, offers the opportunity to understand the processes and timescales through which magmas are stored, differentiated, and delivered prior to eruption (e.g., [5–7, 13, 14, 16, 18, 54, 68, 69]).

**Figure 7:**
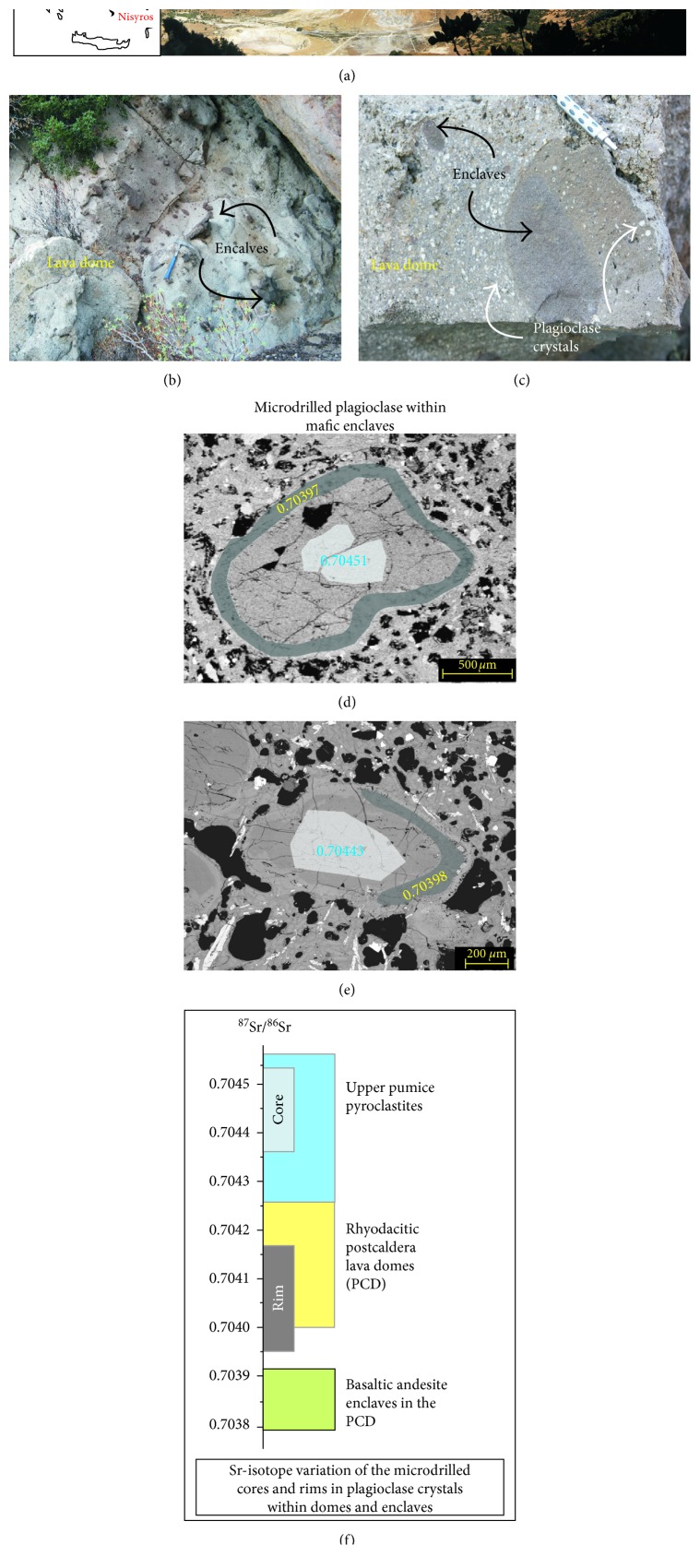
Results of micro-Sr isotope studies on plagioclase crystals from the last magmatic activity of Nisyros volcano (Greece). (a) Landscape view of the Nisyros caldera and its lava domes outpoured during the final magmatic activity of the volcano; (b) image of a lava dome outcrop rich in magmatic enclaves. Notably, the enclaves occurs as well-defined body with rounded and smooth surfaces; (c) specific image of a lava dome and enclave, both rich in large plagioclase crystals; (d and e) back-scattered electron microscope images of two representative plagioclase crystals selected for micro-Sr investigation with microdrill. The shaded areas represent the crystal zones drilled for Sr-isotope analyses, both on cores and rims; (f) ^87^Sr/^86^Sr variation of the drilled cores and rims compared to the range of ^87^Sr/^86^Sr of host whole rock (domes and enclaves) and upper pumice whole rock.

The case study presented here is related to the active Nisyros volcano, the easternmost volcanic island of the South Aegean Active Volcanic Arc (Figure 7) [5, 70–76]. Nisyros volcanic products are typically porphyritic rocks, with clear petrographic evidence of recurrent mixing and mingling of different magmas during the whole volcano's history, which is likely interpreted as the triggering mechanism for its eruptions (e.g., [74, 77, 78]). Sr-isotope determinations at the subcrystal scale, along with detailed petrographic microscopic textural evidence, provided significant data for better defining the interaction of different magmas, concerning pre-eruptive mechanisms. The study focused, in particular, on postcaldera, rhyodacitic dome magmas of the final Nisyros activity emplaced after the caldera-forming rhyolitic explosive eruption of upper pumice. These rhyodacitic lavas contain magmatic enclaves (Figures 7(a) and 7(c)), with basaltic andesite to andesite compositions interpreted, based on their textural features, as quenched portions of mafic magmas included in the cooler, more evolved rhyodacitic host melt (Figures 7(b) and 7(c)) (e.g., [8, 78]).

In situ Sr-isotope ratios were determined on plagioclase phenocrysts (Figures 7(c)–7(e)), from both domes and enclaves, which preserve evidence of the complex history of interaction between the mafic (i.e., enclaves) and felsic (i.e., rhyodacitic domes) magmas in their growing zones [8]. The ^87^Sr/^86^Sr values determined on micromilled samples from the different growth zones of the plagioclase phenocrysts show clear Sr-isotope disequilibria between (i) cores and rims of single crystals (Figures 7(c)–7(e)) and (ii) the crystals and the host magmas (Figure 7(e)). This suggests that some of the phenocrysts that had formed in the rhyolitic magmas were later enclosed (as xenocrysts) within the more mafic one (i.e., as enclaves). Whereas the rim of the phenocrysts is isotopically intermediate between the rhyolitic and mafic magmas (Figure 7(f)), their cores show higher ^87^Sr/^86^Sr values, quite close to that of the previously erupted upper pumice magma. This clearly indicates that the phenocrysts originated in a different, older system. In this light, the large plagioclase phenocrysts found inside the dome lavas and enclaves can be interpreted as recycled from previously cumulated crystals (called “antecrysts” by Davidson et al. [7]).

These results have also demonstrated that the dome lavas are multicomponent magmas formed by progressive mingling/mixing processes between (i) a rhyolitic, and more Sr-radiogenic melt derived from the original upper pumice magmas, and (ii) the enclave-forming mafic, and less Sr-radiogenic, melts refilling the felsic magma chamber.

The constraints involved in interpreting in situ isotope data have further implications for the timing and style of eruption. The inferred delay between the mafic input (i.e., enclaves) and the relative dome eruption allows time for reheating and consequent drop in magma viscosity, thus favouring dome extrusion rather than explosive activity [8, 78].

### 3.2. Micro-Sr Isotope in Natural Glasses

Glasses are found in nature generated by rapid quenching of molten material. They represent a volumetrically small component of crustal rocks and can have different genesis (i.e., volcanic, lightning strikes, meteorite impact, and anthropogenic). In this light, the radiogenic isotopic compositions (i.e., Sr, Nd, and Pb) can provide fundamental information to discriminate among the processes involved in their formation. Glasses constitute the main component of ash and pyroclastic deposits, and their composition and Sr-isotope signature can provide important information in defining the triggering mechanisms of explosive eruptions (e.g., [10, 69, 79–84]). Glasses may also be found in ceramics, as well as in other artefacts. Sr-isotope data are therefore also important to define the possible source of raw materials for pottery, which is particularly relevant for cultural heritage, or to track trade routes in archaeology (e.g., [85–88]). Microscopic scale Sr-isotopic measurements on glasses would help in minimising the amount of sample that needs to be milled, which is of crucial importance both dealing with small-sized volcanic ashes and ejecta and with human artefact of archaeological interest.

**Figure 8:**
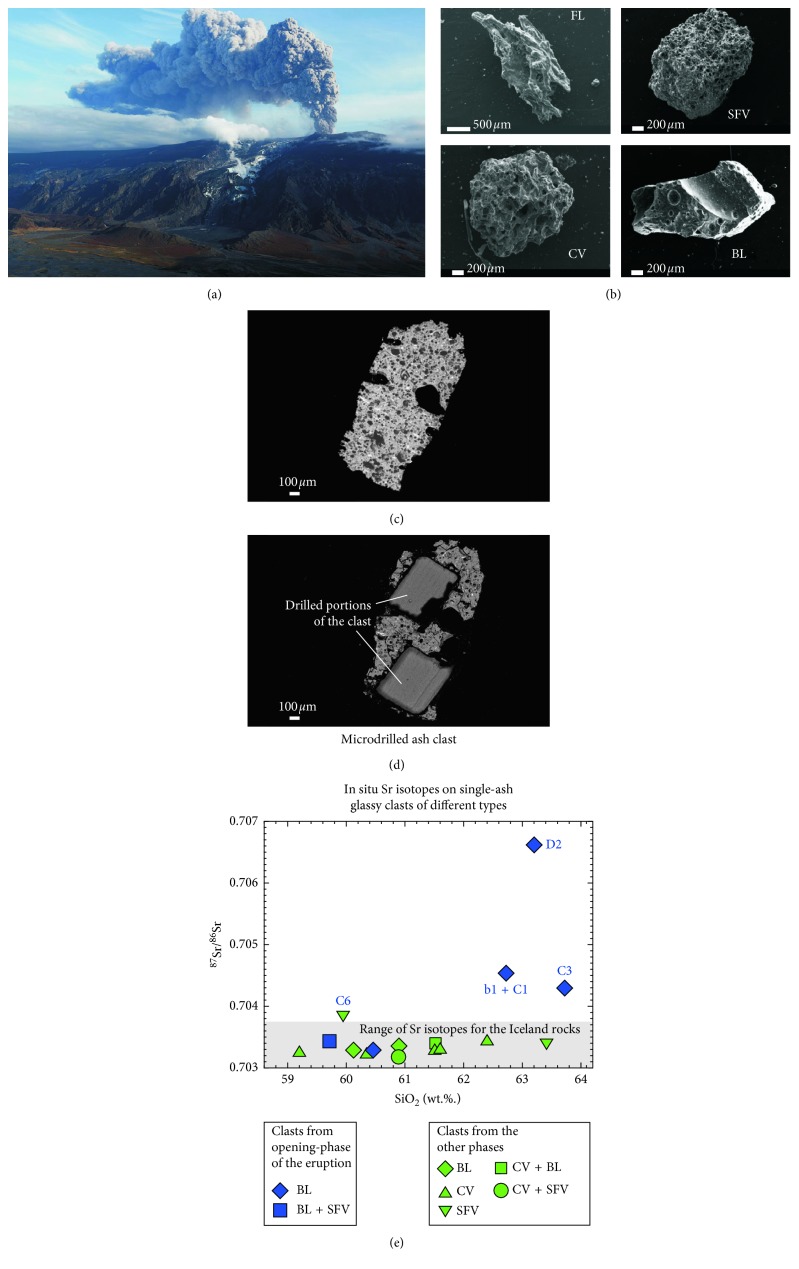
Results of micro-Sr isotopes in volcanic ash glasses of the 2010 explosive eruption of Eyjafjallajökull volcano (Iceland). (a) Spectacular image of the ash cloud erupted by the volcano during the early stage of the 2010 activity (https://hiticeland.com/places_and_photos_from_iceland/eyjafjallajokull causing); (b) back-scattered electron microscope images of different clast types sampled in the basal level of the fallout deposit related to the open phase of the 2010 eruption. FL = fluidal; SFV = spongy finely vesicular; CV = coarse vesicular; and BL = blocky. Scale bars are in micron; comparison between back-scattered electron microscope images of clasts before (c) and after (d) in situ microdrilling. Scale bars are in micron; (e) ^87^Sr/^86^Sr versus SiO_2_ (wt.%) diagram of matrix glasses sampled from single, glassy ash clasts. In situ microsampling for Sr-isotope determinations on glass and plagioclase have been obtained by microdrilling technique. Error bars are inside the symbols.

The present study is a case study of submicroscopic scale Sr-isotopic determination on ashes of the 2010 Eyjafjallajökull (Iceland) volcano's explosive eruption, which caused enormous disruption to air travel across the northern hemisphere (Figure 8(a)) (e.g., [89–94]). This eruption represents a unique opportunity to test the potential of microscale Sr-isotope determinations on ash glasses from tephra deposits that were well preserved within the ice/snow pack [10]. The population of tephra is composed by four different types of ash fragments: (i) fluidal, (ii) coarsely vesicular, (iii) spongy fine vesicular, and (iv) blocky (Figure 8(b); [93]), generated by different fragmentation processes during the eruption. A detailed microanalytical geochemical and in situ Sr-isotope study performed on glassy groundmass of single ash clast showed unusually high ^87^Sr/^86^Sr values (up to 0.70668) for Icelandic volcanism (0.7026–0.7037 [94]) (Figure 8(c)); these high isotopic values were also associated to atypical elemental compositions compared to most of the juvenile ash fragments of the eruption. The anomalous, high Sr-radiogenic clasts belong to the blocky type (Figure 8(b)) and are concentrated in the first, thin ash level emplaced during the initial phase of eruptive activity. These clasts originated from the magma quenched from the contact with the ice cap filling the summit caldera of the volcano [10]. These anomalous findings in the Icelandic magmatic environment can be explained supposing that during its rise and before intruding into the ice cover, the erupting magma selectively assimilated hydrothermal minerals (i.e., zeolites, silica phases, and anhydrite) with seawater-related high-Sr isotopic ratios, hosted in altered volcanic/epiclastic rocks. This selective assimilation took place at the tip edge of the first rising magma body, resulting in a high degree of contamination restricted to the rather small amount of melt directly in contact with the hydrothermal veins. Indeed, evidence for this process is recorded only by the very first erupted juveniles (i.e., the blocky clasts). The results obtained through submicroscopic-scale micromilling and relative Sr-isotope determination revealed the dynamics of the processes involved in the initial stages of magma ascent to the surface; this provides significant insights into the interpretation of the precursory signals of the eruption (mostly consisting of ground deformation or increased seismicity) [10]. These transient processes, which interested only a small, well-confined part of the magma, cannot be detected using traditional, Sr-isotope determination on whole-rock samples but can be revealed only analysing single-glassy clasts separately.

### 3.3. Micro-Sr Isotope in Teeth and Bones

Due to their similar chemical properties, Sr can substitute for Ca in the bioapatite [Ca_5_(PO_4_,CO_3_)_3_(OH,F)] of mammalian bones and teeth, reaching contents of few hundreds of ppm that allows the isotope analysis by microdrilling. The Sr isotope composition of human and animal hard tissues is a function of their dietary habits (e.g., [22]) and depends on the isotopic composition of the food and water ingested during life, which in turn are related to the geological substrate [95–97].

Sr isotopes have been successfully used, along with other stable and radiogenic isotope systematics (i.e., *δ*^13^C and *δ*^18^O and Pb isotopes), not only to track the source regions of migrants and migration pathways, as well as the hunting and trading areas of human populations, but also to study and define the dietary habits of humans and animals (e.g., [23, 44, 95–106]).

A pioneering study [107] demonstrated that migrant individuals who moved between different geologic regions might be traced by comparing ^87^Sr/^86^Sr in adult tooth enamel, formed between four and twelve years of age, and in the bones, which remodel throughout life and therefore representative of adulthood [30]. Unlike bones and dentine, dental enamel formed during childhood [108] remains unaltered throughout the years. Different ^87^Sr/^86^Sr in the teeth and bones of an individual may thus reflect the fact that it moved around the landscape passing through different isotopic environments during its youth and maturity [109, 110]. Teeth enamel is generally preferred to dentine and bones in the analysis of Sr concentrations and isotope ratios because it is virtually unaffected by postmortem diagenesis (e.g., [111–113]).

The micro-scale Sr-isotope measurements of samples obtained using the submicroscopic-scale micromill technique is perfectly able to discriminate between enamel and dentine in single-tooth samples. In addition, this technique increases the accuracy of sampling and also reduces the amount of specimen to be destroyed for high-precision Sr-isotope analysis.

*Ursus spelaeus* was an endemic, widespread European Late Pleistocene species. In contrast to many other taxa, it has fairly rich fossil records, especially thanks to its recurrent use of caves or shelters for winter hibernation [114–116]. Caves were frequently used by bears for many generations, and numerous individuals eventually died in them, so that significant quantities of their remains accumulated over considerable periods of time.

Many studies used stable isotopes to determine the dietary habits of living and extinct bears (e.g., [117–121]), but until now only a small number of papers have considered employing Sr isotopes to possibly elucidate the factors influencing habitat use and gain insights into the foraging behaviour of cave bears [122]. We report here the first ^87^Sr/^86^Sr data obtained through in situ microsampling on teeth and bones of *Ursus spelaeus* found in Grotta all'Onda cave. The study was aimed at defining the lifestyle and feeding behaviour of one of the most prominent European Late Pleistocene mammals [123–125].

**Figure 9:**
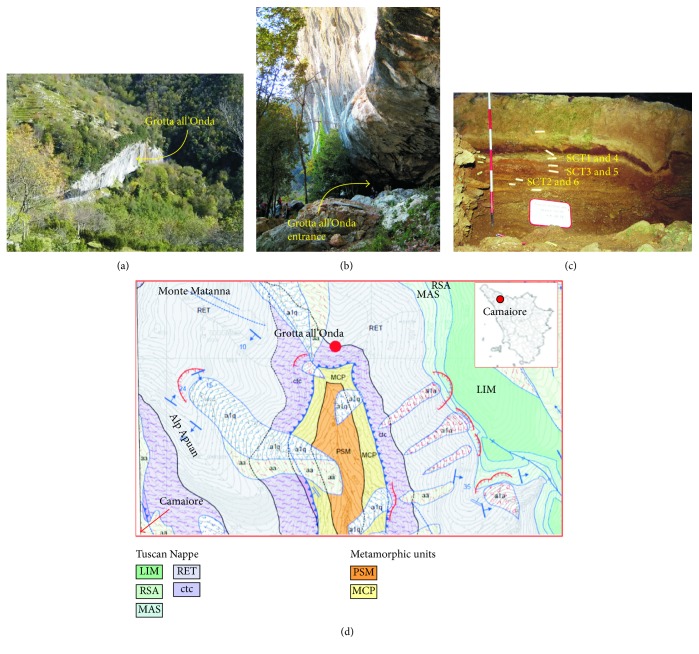
The *Ursus spelaeus* finding environment. (a) Image of the Grotta all'Onda cave from the above, showing the well-defined cut of the Mt. Matanna flank where the cave is located; (b) image of the entrance of the Grotta all'Onda cave; (c) image of the soil stratigraphy from which the *Ursus spelaeus* fossil remains have been sampled during the 1999 field campaign; (d) geological sketch map of the area around the Grotta all'Onda cave (http://www502.regione.toscana.it/geoscopio/geologia.html#). Formation of the Tuscan Nappe unit: LIM: “*Calcare Secifero di Limano*” formation; RSA: “*Rosso Ammonitico*” formation; MAS: “Calcare Massiccio” formation; RET: “*Raethavicola Contorta*” formation, Late Triassic dolomitic-limestones; ctc: cataclastic formation made by a polygenic breccias of mainly metamorphic limestone clasts. Tuscan metamorphic units: PSM: “Pseudomacigno” formation; MCP: “Cipollini” formation.

**Figure 10:**
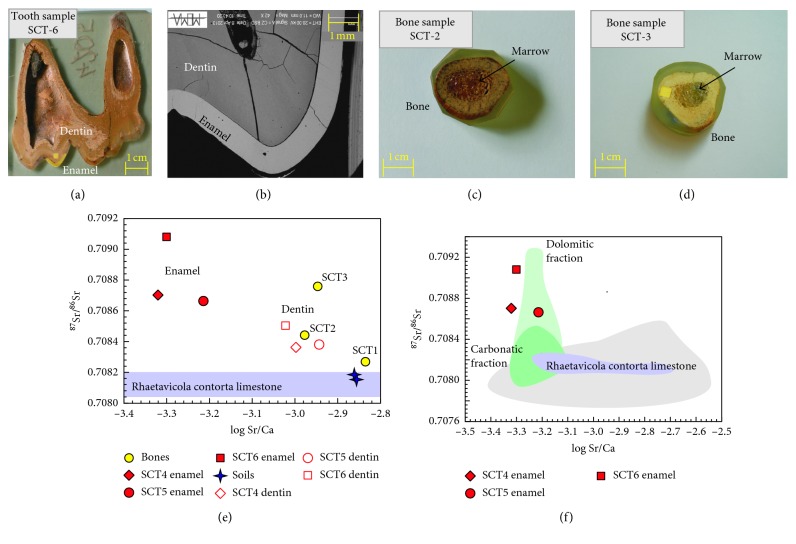
Results of micro-Sr isotopes in teeth and bones of the *Ursus spelaeus*. (a) Representative image of one tooth sample, sectioned for microdrilling. The thin layer of enamel is well evident with respect to the lighter inner dentin. In yellow are reported the two drilling sites on dentin and enamel; (b) particular back-scattered electron microscopy image of the edge of the tooth showing dentin (dark grey) and enamel (light grey) portion; (c and d) images of two of the analysed bones properly prepared in epoxy resin mounts for microdrilling sampling. In yellow is reported the drilling site; (e) ^87^Sr/^86^Sr versus Sr/Ca (in log scale) diagram showing the results obtained from the analyses of enamel and dentin in the three teeth, compared with bones of the *Ursus spelaeus* specimen. The ^87^Sr/^86^Sr of soils in which the fossils have been sampled are also reported together with the Sr-isotope field of the *Raetavicola Contorta* limestone for comparison. (See text for detail) (f) ^87^Sr/^86^Sr versus Sr/Ca (in log scale) diagram comparing the Sr-isotope values of dentin from the three teeth samples with the Sr-isotope range fields of the different geologic formations forming the bedrock outcropping in the area of the Grotta all'Onda cave.

*Grotta all'Onda* cave is located 708 m above sea level (a.s.l.) in the Apuan Alps nearby the village of Camaiore (Tuscany, Italy) (Figure 9), in a sub-Mediterranean habitat [126]. The cave opens at the base of the Tuscan Nappe, at the contact between the “Calcare a Rhaetavicula Contorta Formation” (i.e., Upper Triassic dolomitic-limestone of the Tuscan Nappe) and the “Argilliti Varicolori Formation” (i.e., Lower Cretaceous shales) (Figure 9). The Rhaetavicula Contorta Formation is a polygenic breccia, mainly including metamorphic clasts, known as “Brecce di Grotta all'Onda” (Figure 9) (http://www502.regione.toscana.it/geoscopio/geologia.html). The fossil remains of *Ursus spelaeus* were recovered during a 1999 excavation. Radiocarbon dating of bone yielded ages ranging from 38.22 to 38.28 ky (BP) [127]. Six different specimens were selected for Sr isotope analysis; these include three lower molars (SCT4, SCT5, and SCT6) from layers 7J4, 7J3, and 7J5, respectively and three metapodial bones, SCT 1 (third metacarpal), SCT 2 (fourth metatarsal), and SCT3 (fourth metatarsal), from layers 7J4, 7J5, and 7J3, respectively (Figures 9(c) and 10(a)–10(b)–10(c)–10(d)). Two whole soil (i.e., cave earth) samples, SCT7 and SCT8, were also collected from the representative layers 7J4 and 7J5 (Figure 9(c)).

All the three teeth had well-preserved dentin and very thin enamel layer (Figure 10). In contrast, the three bones were rather differently preserved and had different porosity. In particular, SCT3 was the most heavily mineralised and best preserved, whereas SCT1 was densely vacuolated and preserved higher amounts of organic components.

Major element analyses of these specimens revealed that the enamel bioapatite was more mineralised than that of dentin and bones. Moreover, the dentin bioapatite was found enriched in Sr and in other trace elements [128].

For the purpose of this study, dentin and enamel of the teeth and cortical sections of the bones from Grotta all'Onda were micromilled and analysed for micro-Sr isotope determination. The two soil samples were also processed for Sr-isotope determination using the traditional, large sample method [37, 55]. The ^87^Sr/^86^Sr data are reported in Table 4. Tooth enamel shows higher ^87^Sr/^86^Sr and lower Sr/Ca than microsamples of dentin and bones do (Figures 10(e) and 10(f)). This strongly suggests that ^87^Sr/^86^Sr of tooth enamel is unaffected by diagenetic alteration, in contrast to the other organic-rich samples (i.e., bones and dentin). The ^87^Sr/^86^Sr composition of the dentin samples is close to that of the local soil samples (Figure 10(e)); even closer to the latter is that of the bone samples, with the only exception of SCT3 due to its high degree of mineralisation.

These results, despite the relatively recent age of the fossil specimens (ca. 40 ka [127]), indicate that bone tissues have been more exposed to *postmortem* diagenetic exchange processes compared to more heavily mineralized enamel. This speaks for a possible isotope reequilibration between dentin and bones (but not enamel) and soil, due to Sr exchange with percolating fluids. The soil samples have ^87^Sr/^86^Sr values comparable to those of the “Calcare a Rhaetavicula Contorta” formation, which forms the cave's bedrock [129], through which fluids filter into the cave (Figure 10(e)).

In summary, our study shows that the Grotta all'Onda bones and dentin are unsuitable to determine the characteristics of the habitat where the cave bear lived, due to their interaction with percolating water and their consequent contamination by the soils in which they had been preserved. In contrast, the ^87^Sr/^86^Sr ratios of the tooth enamel results unaltered and realistically reflects the original values achieved during the cave bear's life. The isotopic composition of the enamel samples is consistent with that of the “Calcare Massiccio Formation” and in particular with the dolomite fraction [129] (Figure 10(f)). The mismatch between the ^87^Sr/^86^Sr values of the enamel samples and those of the cave soils (Figure 10(e)) (i.e., local substrata of the cave) indicate that the cave bear died away from its customary habitat. Bears cannot indeed find food in caves, where they find refuge as shelter for winter hibernation or for night resting. The enamel isotopic values obtained during our study indicate that *Ursus spelaeus* from Grotta all'Onda roamed in search for food within a confined area not far from the cave, where the “Calcare Massiccio” is largely exposed and did not move too far from the area during its whole life.

## 4. Summary and Conclusions

The present study show the potential of ^87^Sr/^86^Sr determination by TIMS on micro-scale samples, based on micromilling solid specimens, not only for geological applications, but also for other fields, such as archaeology, forensics, medical, and life sciences, where it has hardly, if ever, been used. Reported here is a detailed description of all the analytical protocols, including results on replicate analyses of international standards (SRM 987 and BHVO-2G), which yield good accuracy and precision. In addition, three case studies are presented, performed in our laboratory, where in situ microdrilled Sr isotopes have been used in different fields of application.

The first case study on micro-Sr isotope determination at subgrain microscopic scale regards the petrogenetic processes relevant to the understanding of the plumbing system dynamics under active volcanoes. This example revealed the role played by the interaction of different magmas, which are normally characterised by distinct ^87^Sr/^86^Sr signatures, comingled in the plumbing system of the Nisyros volcano, which was capable of triggering the eruption.

The second case regards volcanic glasses with extremely low total Sr content (i.e., tholeiitic). The micromilling determination of ^87^Sr/^86^Sr ratio was performed on ashes, with different shapes and nature, erupted by different phases, during the 2010 eruption of Eyjafjallajökull volcano (Iceland). The ^87^Sr/^86^Sr data provide information on the eruptive mechanism involved during the eruption, as well as on the interaction between the magma and the hydrothermally-derived minerals attained before the thawing of the ice cap; it also provides significant insights into the interpretation of the precursory signals of the eruption.

The third case displays the use of ^87^Sr/^86^Sr microdrilled in enamel, dentin, and bones, to show that only enamel has more chances to preserve the original Sr-isotope signatures than bone and dentin. The analysis also revealed the close relationship existing between the radiogenic-Sr of the organic materials and that of the geologic Cretaceous substratum of the Apuan Alps, which provides valuable insights into the palaeoenvironment of the local cave bears. In this case, the Sr isotopes proved particularly useful for determining the foraging habits of extinct mammals, which substantiates the well-known statement “YOU ARE WHAT YOU EAT” (cit. Anthelme Brillat-Savarin).

## Figures and Tables

**Table 1:** Blank contamination level.

	Laboratory blanks			Isotope ratios on blanks		
*Standard procedure on large size samples*	Sr pg	1 SD	*n*	^87^Sr/^86^Sr	1 SD	*n*
	127	60	32	0.707497	0.000060	5
*Procedure on small size samples*	38	19	16			
Total procedure (drilling, digestion, and elemental selection)						
Chemical digestion and separation	17	6	12			

1 SD, standard deviation (external precision); *n*, numbers of blank measurements; the average Sr-isotope value obtained from 5 unspiked blanks is reported to fully characterize the potential contamination component.

**Table 2:** Cup configuration schemes of static (a) and dynamic (b) mode measurements.

Cup	L4	L3	L2	L1	C (Far)	H1	H2	H3	H4
*(a) Static collection mode*									
		^84^Sr	^85^Rb	^86^Sr	^87^Sr	^88^Sr			
*(b) Dynamic collection mode*									
Jump 1			^85^Rb	^85^Rb	^86^Sr	^87^Sr	^88^Sr		
Jump 2 (main)		^84^Sr	^86^Sr	^86^Sr	^87^Sr	^88^Sr			
Jump 3				^87^Sr	^88^Sr				
*Sr double*									
Combining the measurements from two different magnetic field position (jump 1-2 and jump 2-3 above), it is possible to evaluate two independent ^87^Sr/^86^Sr_double_ defined as follows:Sr87/Sr861-2=Sr87H1/Sr86C1·Sr87C/Sr88H12·Sr88/Sr86N and Sr87/Sr862-3=Sr87C/Sr86L12·Sr87L1/Sr88C3·Sr88/Sr86N. The numbers outside the parentheses are relative to the three different magnetic field positions, the subscript of each isotope refers to the cup on which it is measured, and [^88^Sr/^86^Sr]_*N*_ is the natural ratio (i.e., 8.375209)									

**Table 3:** Accuracy and reproducibility on reference standard material (NIST-SRM987 and BHVO-2).

Standard	Sr content	^87^Sr/^86^Sr	2 SD	*n*	Reference
*Within run measurement on international standard NIST-SRM987*					
*Large size loading (ng)*					
SRM987	150	0.710253	0.000016	59	Long-term reproducibility (from 2013)
SRM987	150	0.710248	0.000011	427	Thirlwall [59]
*Small size loading*					
SRM987	10	0.710252	0.000016	47	This study
SRM987	12	0.710259	0.000018	92	Charlier et al. [54]

*Drilling procedure on BHVO-2 glass*					
BHVO-2 glass	<10	0.703490	0.000092	9	This study
BHVO-2 glass	10	0.703492	0.000094	3	Charlier et al. [54]
		^87^Sr/^86^Sr	2 SE		
BHVO-2 powder	150	0.703469	0.000004	1	This study
		^87^Sr/^86^Sr	2 SD		
BHVO-2 powder	—	0.703479	0.000020	12	Weis et al. [60]

2 SD, two standard deviation (external precision); 2 SE, two standard error of the mean; *n*, numbers of measurements; literature data are reported in italics below our mean values for references.

**Table 4:** (a) ^87^Sr/^86^Sr results obtained on microsamples of tooth dentine and enamel and bones of the *Ursus spelaeus* specimen. (b) ^87^Sr/^86^Sr soils sampled in the Grotta all'Onda cave from which the fossil remains were collected.

Sample	Type	Sr content (ppm)	^87^Sr/^86^Sr	2 SE	Log (Sr/Ca)
					Average
*(a) Teeth and bones*					
STC1	Bone	510	0.708268	0.000006	−2.84
STC2	Bone	352	0.708441	0.000006	−2.98
STC3	Bone	359	0.708758	0.000006	−2.95
STC4-1	Enamel	187	0.708703	0.000005	−3.32
STC4-2	Dentin	360	0.708362	0.000006	−3.00
STC5-1	Enamel	211	0.708663	0.000005	−3.21
STC5-2	Dentin	384	0.708380	0.000006	−2.94
STC6-1	Enamel	179	0.709081	0.000005	−3.30
STC6-2	Dentin	339	0.708504	0.000006	−3.02
*(b) Soils*					
STC7	Soil	204	0.708184	0.000006	−2.86
STC8	Soil	228	0.708155	0.000006	−2.86

2 SE: two standard error of the mean.
